# Molecular Characterization of the Predominant Influenza A(H1N1)pdm09 Virus in Mexico, December 2011–February 2012

**DOI:** 10.1371/journal.pone.0050116

**Published:** 2012-11-29

**Authors:** Daniela de la Rosa-Zamboni, Joel A. Vázquez-Pérez, Santiago Ávila-Ríos, Ana Paola Carranco-Arenas, Christopher E. Ormsby, Craig A. Cummings, Maribel Soto-Nava, Víctor A. Hernández-Hernández, Carmen O. Orozco-Sánchez, Claudia Alvarado-de la Barrera, Rogelio Pérez-Padilla, Gustavo Reyes-Terán

**Affiliations:** 1 Instituto Nacional de Enfermedades Respiratorias Ismael Cosío Villegas, Mexico City, Mexico; 2 Life Technologies Corporation, Foster City, California, United States of America; University of Hong Kong, China

## Abstract

When the A(H1N1)pdm09 pandemic influenza virus moved into the post-pandemic period, there was a worldwide predominance of the seasonal influenza A(H3N2) and B viruses. However, A(H1N1)pdm09 became the prevailing subtype in the 2011–2012 influenza season in Mexico and most of Central America. During this season, we collected nasopharyngeal swabs of individuals presenting with influenza-like illness at our institution in Mexico City. Samples were tested for seasonal A(H3N2) and B influenza viruses, as well as A(H1N1)pdm09 by real-time reverse transcription–polymerase chain reaction. Of 205 samples tested, 46% were positive to influenza, all of them A(H1N1)pdm09. The clinical characteristics of patients showed a similar pattern to the 2009 pandemic cases. Using next generation sequencing, we obtained whole genome sequences of viruses from 4 different patients, and in 8 additional viruses we performed partial Sanger sequencing of the HA segment. Non-synonymous changes found in the Mexican isolates with respect to the prototype isolate H1N1 (A/California/04/2009) included HA S69T, K163R and N260D unique to 2012 Mexican and North American isolates and located within or adjacent to HA antigenic sites; HA S143G, S185T, A197T and S203T previously reported in viruses from the 2010–2011 season, located within or adjacent to HA antigenic sites; and HA E374K located in a relevant site for membrane fusion. All Mexican isolates had an oseltamivir-sensitive genotype. Phylogenetic analysis with all 8 influenza gene segments showed that 2012 Mexican sequences formed a robust, distinct cluster. In all cases, 2012 Mexican sequences tended to group with 2010–2011 Asian and European sequences, but not with 2009 Mexican sequences, suggesting a possible recent common ancestor between these latter regions and the 2012 Mexican viruses. It remains to be defined if these viral changes represent an important antigenic drift that would enable viral immune evasion and/or affect influenza vaccine effectiveness.

## Introduction

The pandemic A(H1N1)pdm09 influenza virus was originally detected in Mexico and the United States in March 2009 [1]. Three months later, the World Health Organization announced that an influenza pandemic was underway. In August 2010 the global alert was moved into the post-pandemic period, but localized outbreaks of various magnitudes were likely to continue [2]. In the United States during the 2010–2011 influenza season, influenza viruses B, A(not subtyped), A(H3) and A(H1N1)pdm09 were observed circulating in similar proportions; in Mexico the predominant subtype was A(H3) and only small proportions of influenza viruses B, A (not subtyped) and A(H1N1)pdm09 were detected [3]. During the 2011–2012 influenza season in the United States, the aforementioned influenza viruses were still circulating but the predominant subtype was A(H3); in Mexico, a contrasting predominance of the A(H1N1)pdm09 virus was observed, with very limited circulation of influenza viruses A(H3) and B [3], [4]. Furthermore, this A(H1N1)pdm09 virus predominance during the 2011–2012 influenza season was also observed in Guatemala, Honduras, El Salvador, Nicaragua, Costa Rica and Panama [3]. Thus, distinctive patterns of the 2011–2012 influenza season were observed within North and Central America, with predominance of the A(H3) subtype and B lineage in the United States and Canada, respectively, and a predominance of the A(H1N1)pdm09 subtype in Mexico and most of Central America. The temporal relationship between the last influenza season in Mexico (from epidemiological week 47, 2011 to week 13, 2012) and Guatemala (from week 7, 2012 to week 10, 2012) suggests that the A(H1N1)pdm09 virus spread from Mexico to Guatemala and perhaps to southern regions.

In other parts of the world, A(H1N1)pdm09 was a minority variant in the 2011–2012 season, although it appeared to be associated with more severe clinical cases [5].

As part of a continuous surveillance program implemented at The National Institute of Respiratory Diseases (INER; a national referral center for respiratory diseases treating mostly uninsured patients in Mexico City), patients presenting with influenza-like illness (ILI) were tested for the seasonal as well as for the pandemic influenza virus A(H1N1)pdm09. Between December 24, 2011 and February 14, 2012, a marked predominance of the A(H1N1)pdm09 virus was observed. The Center for Research in Infectious Diseases at the INER conducted an investigation to describe this outbreak and to characterize the virus. Here we present the first virological characterization of the predominant A(H1N1)pdm09 virus in the region during the 2011–2012 influenza season, and discuss the potential impact of observed genetic polymorphisms on viral pathogenicity and influenza vaccine effectiveness.

## Materials and Methods

### Ethics Statement

All tests and sampling in this study were carried out for institutional diagnostic and epidemiological purposes. All hospitalized patients gave written informed consent to sample donation and to allow diagnostic tests to be performed. The Science and Bioethics Committee of the National Institute of Respiratory Diseases (INER) revised and approved this consent procedure. Ambulatory patients gave verbal informed consent to diagnostic procedures. The institutional epidemiology department formally documented the process, including patient reception, initial evaluation, data collection, sampling, diagnostic testing and treatment, in agreement to institutional proceedings and with acceptance of the Science and Bioethics Committee of the INER. For all pediatric patients, the corresponding legal guardians provided written consent. Sampling for pediatric patients was treated separately within a special research protocol revised and approved by the Science and Bioethics Committee of the INER. Individual data were kept anonymous in all cases.

### Sample Collection, RNA extraction and influenza detection

As part of a surveillance program at the INER, nasopharyngeal swabs of individuals presenting with ILI are routinely collected. Patients with acute respiratory disease or chronic lung disease exacerbation requiring hospitalization, as well as ambulatory health workers with ILI, were considered as suspect cases. Bronchial aspirates from patients requiring mechanical ventilation were also tested. Viral RNA was extracted from 200 µl samples using a high throughput system (MagNA Pure, Roche, Indianapolis, IN, USA) and tested for seasonal A(H3N2), B, and pandemic A(H1N1)pdm09 influenza viruses by real-time reverse transcription–polymerase chain reaction (rRT-PCR), using in-house designed primers that were cross-checked in accordance with guidelines from the U.S. Centers for Disease Control and Prevention (CDC). Cases were considered to be confirmed if the A(H1N1)pdm09 virus was detected. Demographic and clinical data were collected through review of clinical charts and during sample collection.

### Hemagglutinin partial sequencing

Partial sequencing of the HA segment was performed in 8 patients, directly form clinical samples. Briefly, 1,583 bp amplicons were obtained using specific primers for A(H1N1)pdm09: HA 58F 5′ TTATGTATAGGTTATCATGCGAA 3′ and HA 1676R 5′ ACCCATTAGAGCACATCCAGAAAC 3′ in 25 µL reactions using the OneStep RT-PCR Kit (QIAGEN, Valencia, CA). The following PCR conditions were used: 50°C, 30 min; 95°C, 2 min; 45 cycles (95°C, 15 s; 55°C 30 s and 72°C, 30 s); 72°C, 5 min. A 2.5 µL aliquot of the first reaction was then used as template in a second round of reactions with HA 149F 5′ TAGAAGACAAGCATAACGGGAAA 3′ and HA 865R 5′ CTGGTGTATCTGAAATGATAATA 3′ (608 bp); or HA H1F562 5′ AAATCCTACATTAATGATAAAGGGA3′ and HA H1R 1319 5′ GCATTGTAAGTCCAAATGTCCA 3′ (782 bp), using Platinum Taq DNA Polymerase High Fidelity (Invitrogen, Carlsbad, CA). The following PCR conditions were used: 95°C, 2 min; 35 cycles (95°C, 15 s; 55°C, 30 s; 72°C, 30 s); 72°C, 5 min. The second round amplicons were sequenced in both directions with the latter primers. Sequencing reactions were performed with BigDye Terminator v3.1 (Life Technologies, Carlsbad, CA) as instructed by the manufacturer. Sequences were obtained by capillary electrophoresis using an ABI Prism 3130 Genetic Analyzer (Life Technologies) and were assembled using BioEdit V7.0.5.3 and MEGA 5.0. The aforementioned sequences can be found at GenBank (accession numbers JQ934940–JQ934947).

### Influenza Virus Whole Genome Sequencing

Whole genome sequences of viruses from four patients were obtained by semiconductor next generation sequencing (NGS), using an Ion PGM™ Sequencer (for research use only, not for diagnostic purposes, Life Technologies, Carlsbad, CA). These four viruses were obtained from different patients than those in which Sanger sequencing was performed with the only selection criteria of having a high viral load to facilitate amplification and sequencing. The 8 viral segments were amplified simultaneously and directly from clinical samples, using a multi-segment RT-PCR with MBTuni12 and MBTuni13 primers, as described elsewhere [6]. The viruses were not passaged in cells or eggs before sequencing. Amplification products for 3 samples were gel-purified (QIAquick Gel Extraction Kit; QIAGEN, Valencia, CA) in two pools: one containing PB2, PB1 and PA and another containing HA, NP, NA, M and NS. The amplification product for the remaining sample was directly purified using the QIAquick PCR Purification Kit (QIAGEN). Barcoded libraries for NGS were produced for each pool separately, according to standard protocols, using the Ion Xpress Plus Fragment Library Kit (Life Technologies). The barcoded libraries were pooled in equimolar rates and template for sequencing was produced manually. Sequencing was performed on an Ion 316™ chip according to standard protocols, using the Ion Sequencing 100 bp Kit (Life Technologies). The reads were mapped to the A/California/07/2009(H1N1) reference (accession numbers FJ981613, FJ969527–FJ969531, GQ338390 and GQ377078) using TMAP V.0.2.3 and further analysis was carried out using SAMtools [7]. A 100% coverage was achieved for each virus, with at least 60× depth for all viral segments (mean coverage: 3,583×) in the three gel-purified samples, and in the HA, NS, NA, M and NP segments of the additional sample sequenced directly from the multi-segment RT-PCR without separating into two pools. The PA, PB1 and PB2 segments of this last sample had 85% coverage at >10×. The consensus public sequences are available in GenBank (accession numbers JQ714072–JQ714075 and JQ927301–JQ927328).

### Phylogenetic Analysis

Full-length nucleotide sequences were selected for all segments of human influenza H1N1 viruses reported between 2009 and March 15, 2012 at the Influenza Research Database [8]. A total of 23,513 sequences with 2,939 sequences for each segment were retrieved, except for HA (2,941) and NS (2,938), by selecting only pandemic viruses and including genomes that had all corresponding segments. We included the four whole genome sequences from this study for the analyses of all 8 viral segments. Alignments were created with MAFFT [9] and manually edited with MEGA 5.0.5 [10]. A neighbor-joining (NJ) tree was constructed for each influenza segment using MEGA 5.05. The Tajima-Nei model was selected with 5-parameter gamma distributed rates and 1,000 bootstrap replicates. Further analysis and edition was done with package ape 2.7–3 in R 2.13.2 [11]. Evolutionary divergence analysis between HA sequences was conducted using the Kimura 2-parameter model [12] in MEGA 5.0.5. The analysis included 2,945 nucleotide sequences. Codon positions were 1st+2nd+3rd+ Noncoding.

We then selected subsets of 136 to 162 sequences per genetic segment, including representative sequences from around the world, all Mexican sequences, and sequences that grouped with the 2012 Mexican cluster in the initial NJ phylogeny. Viruses with sequences available for most genetic segments were preferred. Using these subsets, we constructed unrooted maximum likelihood trees with 1,000 bootstrap replicates using the Tajima-Nei model in MEGA 5.05 for each genetic segment. These trees were used for describing the phylogenies.

Distances and frequencies were defined by grouping sequences as 2012 Mexican (sequences obtained in this work), 2009–2011 Mexican, and sequences from their respective influenza transmission zones as defined by the WHO [13].

### Protein Modeling

The three-dimensional structure of the 2012 H1N1 Mexican influenza HA was modeled with the ESyPred3D web server [14] using the 3D structure 3LZG chain ‘A’ as template [15]. Further editing was performed with the UCSF Chimera package [16]. Chimera is developed by the Resource for Biocomputing, Visualization, and Informatics at the University of California, San Francisco, with support from the National Institutes of Health (National Center for Research Resources grant 2P41RR001081, National Institute of General Medical Sciences grant 9P41GM103311).

### Statistical Analysis

Stata 11 was used for the statistical analysis of clinical data. Exact Fisher Tests were used for comparing proportions and Wilcoxon Rank sum tests for comparing medians. In order to analyze factors associated with severity, a model was created using logistic regression. Variables were retained in the model if the likelihood-ratio test p values were less than 0.05.

## Results

### Clinical characteristics of the patients

**Figure 1:**
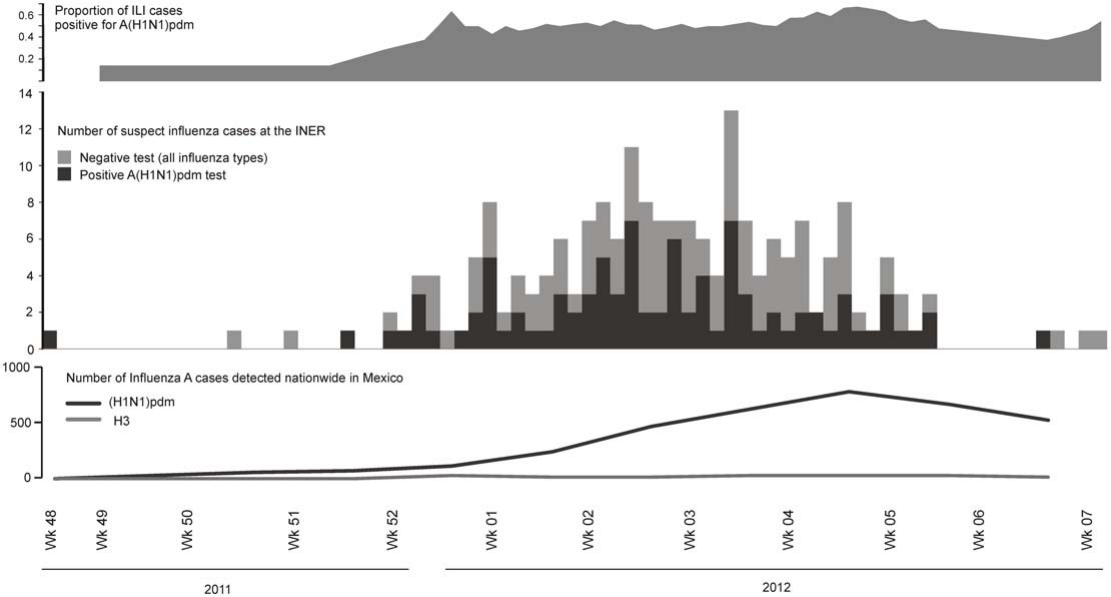
Number of influenza like illness (ILI) cases (middle track) and proportion positive for the A(H1N1)pdm09 virus (upper track, 3 week moving average) during epidemic weeks 48–2011 to 07–2012 at the National Institute of Respiratory Diseases (INER) in Mexico City. Dates are for onset of symptoms. The lower track shows the number of influenza A cases reported in Mexico according to the WHO (http://apps.who.int/globalatlas/dataQuery/default.asp, last accessed on February 27, 2012). Week 7 was omitted due to a lag in reporting to this database.

Between October 4 and December 23, 2011, nasopharyngeal swabs from 90 patients with ILI were obtained and all tested negative for influenza viruses. On December 24, 2011, the A(H1N1)pdm09 virus was first detected in a 9-year-old boy with acute asthma exacerbation. Confirmed and suspect cases increased since the last week of December 2011, when the hospital authorities were informed about a possible reemergence of the A(H1N1)pdm09 virus. During the first week of January 2012, preventive contact measures were intensified. Influenza trivalent vaccination was encouraged for all health care workers and staff at the hospital and was available to anyone upon request. Cases of influenza A(H1N1)pdm09 were already being reported in other hospitals in the south and center of the country. On the third week of January, patients with ILI occupied 40% of the hospitalization beds. By the second week of February 2012, 205 patients (165 hospitalized and 40 ambulatory) had been tested, and 95 (46%) had been positive for the A(H1N1)pdm09 virus (Figure 1), but no other influenza viruses were detected.

Baseline characteristics and clinical data observed in confirmed and suspect cases were similar (p>0.1) for most parameters analyzed (Table 1). The median age was 39 years (interquartile range (IQR): 26–52), 28 patients (14%) were under the age of 19, and 126 patients (61%) were male. Previous lung diseases were reported by 36 patients (18%). Forty nine patients (24%) were smokers, and 39 (19%) were immunocompromised. The most frequent symptoms were cough in 170 patients (83%), fever in 153 (75%), dyspnea in 130 (63%) and malaise in 120 (58%), with 95% of the patients presenting with one or more of these symptoms. Approximately half of the patients reported myalgia/arthralgia, nasal discharge/congestion and headache. The presence of nasal discharge was significantly more common in confirmed cases than in suspect cases (61, 64% vs. 47, 43%; p = 0.003). This observation could be related to a more efficient detection of the virus in nasopharyngeal samples from the confirmed cases. Nearly all patients (97%) were treated with oseltamivir. Samples were obtained before oseltamivir treatment initiation in 51.3% and 60.0% or within de first 24 h of treatment initiation in 29.0% and 17.6% of the confirmed and suspect cases respectively, with no apparent effect of early treatment on virus detection (p = 0.7). Forty two patients (20%) required mechanical ventilation. Older age (OR = 1.98, p = 0.02) and cardiovascular disease (OR = 2.88; p = 0.04) were risk factors for mechanical ventilation. Patients requiring mechanical ventilation sought medical care later than the rest of the patients (7 days, IQR 4–10 vs. 4 days, IQR 2–8; p = 0.001). Interestingly, according to medical records, at least 18% of the confirmed cases and 22% of the suspect cases had received influenza vaccination during the 2011–2012 season (Table 1). Given an effectiveness of the A(H1N1)pdm09 vaccine placed at 87.3% [17], and the vaccination coverage in Mexico at 56.53% [18], we could expect 7.18±0.5% (95% CI for 10,000 cases; the number of cases was larger in both references) of vaccinated individuals in the positive influenza population, but in fact we found 20±2.81% (95% CI for 95 cases). It is important to note that the same percentage of vaccinated individuals was observed in both the immunocompromised and immunocompetent groups (20%, p = 1.00). Moreover, no difference was observed between the number of vaccinated immunocompromised and vaccinated immunocompetent confirmed cases (22.2% and 16.9% respectively, p = 0.60).

**Table 1:** Baseline clinical data of the study population.

	Suspect cases [n(%)]^a^	Confirmed cases [n(%)]^b^	p value^c^
n	110(54)	95(46)	
Age (median, IQR)	38(28–54)	39(23–50)	0.25
Male gender	69(63)	57(60)	0.74
2011–2012 Influenza vaccination^d^	25(22)	17(18)	0.49
Oseltamivir treatment	103(93)	93(98)	0.18
Patients hospitalized	87(79)	78(82)	0.60
Patients requiring mechanical ventilation	22(20)	20(21)	0.86
Death	6(5)	4(4)	0.75
Any comorbidity	64(58)	55(58)	1.00
Lung disease	19(17)	17(18)	1.00
Immunocompromised	21(19)	18(19)	1.00
Cardiovascular disease	12(11)	7(7)	0.47
Diabetes	9(8)	5(5)	0.58
Morbid obesity	9(8)	5(5)	0.58
Mean time with symptoms (SD)	6.7 (6.7)	5.7(4.1)	0.22
Symptoms			
-Fever	79(72)	74(78)	0.33
-Cough	86(78)	84(88)	0.06
-Dyspnea	69(63)	61(64)	0.88
-Malaise	60(54)	60(63)	0.25
-Nasal discharge/congestion	47(43)	61(64)	0.003
-Headache	50(45)	50(43)	0.32
-Myalgia/arthralgia	51(46)	48(50)	0.57
-Abnormal lung exploration	33(30)	33(35)	0.55

a Patients presenting with influenza-like illness (ILI), A(H1N1)pdm2009 influenza virus not detected.

b Patients presenting with ILI, A(H1N1)pdm2009 influenza virus detected.

c Chi-square test.

d 9 patients, 4 confirmed and 5 suspect cases ignored vaccination status. Vaccinated cases confirmed by medical records.

### Molecular analysis of hemagglutinin

**Figure 2:**
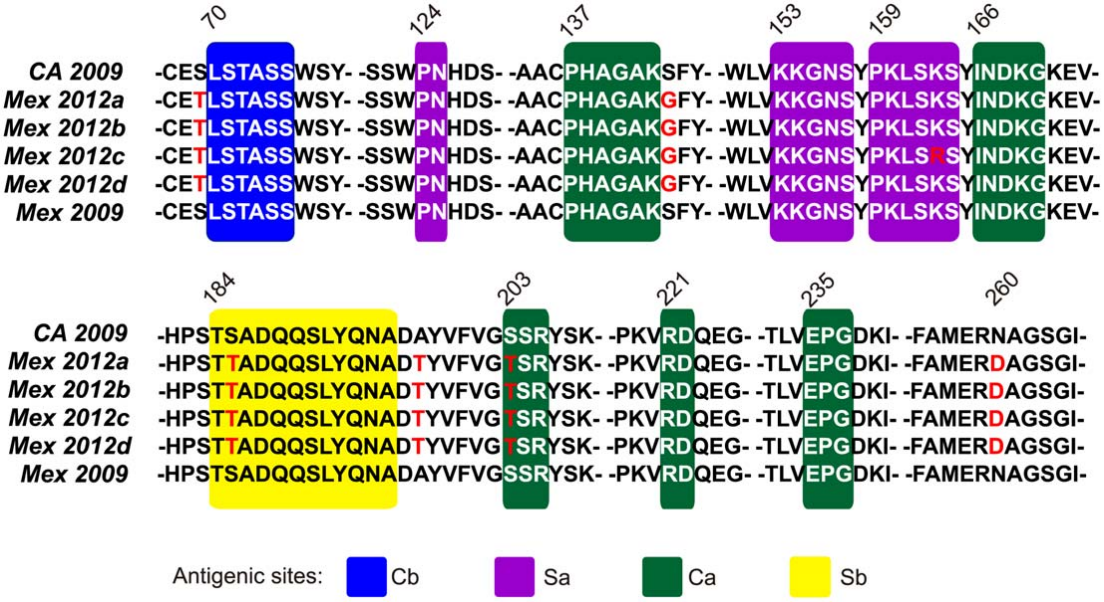
Amino acid substitutions in the HA antigenic sites of the 2012 influenza A H1N1 Mexican isolates. Amino acid sequences are shown for the HA antigenic region of the four fully sequenced 2012 Mexican isolates (MX2012a-d, GenBank accession numbers JQ714072–JQ714075 respectively), the reference California 2009 isolate (CA2009, accession number CY054707) and a reference 2009 Mexican isolate (MX2009, accession number GQ402189). Amino acid positions are numbered without considering the HA signal peptide. Amino acid substitutions in the 2012 Mexican isolates are marked in red. Antigenic sites are shaded: Sa – purple, Sb – yellow, Ca – green, Cb – blue. Substitutions S69T, S143G, K163R, S185T, S203T and A197T are shown to be within or adjacent to antigenic sites.

Non-synonymous changes found in the 2012 Mexican isolates with respect to the prototype isolate H1N1 (A/California/04/2009) are shown in Table 2. Interestingly, HA mutations S69T, K163R and N260D were unique to 2012 Mexican and USA isolates and had not been reported in other circulating viruses before. These changes were found in the four viral genomes obtained by NGS as well as in the 8 partial HA sequences obtained by Sanger sequencing. Similarly to other A(H1N1)pdm09 viruses, Mexican isolates included the HA E374K mutation located in a relevant site for membrane fusion [19]. Mexican isolates maintained the eight putative glycosylation sites at HA positions 27, 28, 40, 104, 293, 304, 498 and 557, and possessed a single basic amino acid at the HA cleavage site instead of the polybasic cleavage site of highly pathogenic H5 viruses. Mapping of substitutions to the known H1 antigenic sites [20] showed that mutation S185T, observed in all 2012 Mexican isolates (but not in 2009 isolates), was located within the Sb antigenic motif; and that mutation S203T, also observed in all the 2012 Mexican isolates, was located within the Ca antigenic site. Similarly, the substitution K163R, observed in only one of the 2012 Mexican isolates, was located within the Sa antigenic site. Moreover, mutations S69T, S143G and A197T were found in the vicinity of the Cb, Ca and Sb sites respectively (Figure 2). Most of these mutations have been previously observed in viruses circulating mainly in Asia, during the 2010–2011 influenza season (Table 2). Nevertheless, S69T and K163R represent novel mutations in 2012 Mexican isolates that are located within HA antigenic sites, with a possible impact in HA antigenicity.

**Table 2:** Non-synonymous mutations in 2012 Mexican isolates with respect to the prototype isolate A/California/04/2009.

Gene	Substitution	Sequences reported worldwide with the substitution (2010–2011 influenza season)*
PB1	T20I	Not reported
	I397M	Sequences from Taiwan, Bangkok, Sydney, Ulaanbaatar, Moscow in the 2010–2011 influenza season
	I435T	Sequences from Taiwan, Bangkok, Sydney, Ulaanbaatar Moscow in the 2010–2011 influenza season, cosegregated with I397M
PB2	V344M	Most of the sequences from the 2010–2011 influenza season
HA‡	S69T	Not reported (only sequences from the USA and Mexico from 2012)
	S143G	Sequences from Taiwan, Bangkok, Sydney, Ulaanbaatar, Moscow in the 2010–2011 influenza season
	K163R†	Not reported
	S185T	Sequences from India, Hong Kong and the UK in the 2010–2011 influenza season
	A197T	Sequences from Hong Kong and Japan
	N260D	Not reported (only sequences from the USA and Mexico from 2012)
	S203T	Sequences from Japan, India, Russia and the UK
	E374K	Sequences from Hong Kong, Singapore, New Zealand and UK
	S451N	Detected and characterized branches and sub-branches in Hong Kong and UK
	V520A	Few sequences in Genebank from Dakar, Finland, Sydney and Moscow
NP	V217I	Few sequences from Asia 2010
NA	G41R	One sequence from Mexico 2010
	V241I	Most of the sequences from the 2010–2011 influenza season
	N369K	Most of the sequences from the 2010–2011 influenza season
M1	V80I	Most sequences from the 2010–2011 influenza season
NS1	L90I	Sequences from Taiwan, Bangkok, Sydney, Moscow, Beijing and Shanghai in the 2010–2011 influenza season
NS2	S60N†	Few sequences from the 2010–2011 influenza season

* Non-synonymous mutations with respect to the prototype isolate A/California/04/2009,(accession number GQ280797.1) are reported. Each polymorphism was searched in a set of 2,939 genome sequences retrieved from GenBank, and selected through FluDB, aligned by Clustal X and analyzed with Mega version 5.0.5. Sequences from viruses circulating in all the geographical transmission zones defined by the WHO worldwide were included [13].

† Substitution detected in one of the four 2012 Mexican isolates.

‡ HA positions are given after removing the signal peptide.

Three-dimensional models of the HA proteins of two 2012 Mexican isolates were predicted based on the crystal structure of a 2009 H1N1 influenza virus HA chain A [15] (PDB 3LZG), in order to explore the possible impact of new substitutions in the already known HA antigenic sites (Figure S1). Readily accessible substitutions associated with antigenic sites were found in the three-dimensional models of the 2012 Mexican HA, such as S143G, K163R, S185T and S203T, which could alter the antigenicity of the protein. Other substitutions such as S69T and A197T seemed to be less exposed. Some substituting amino acids possess similar chemical properties to the 2009 amino acids, arguing against important alterations in charge and hydrophobicity. Nevertheless, a possible influence of these amino acid substitutions on 2012 Mexican HA antigenicity needs to be further studied.

### Associations between genetic polymorphisms and possible pathogenicity of the 2012 Mexican isolates

In order to detect other mutations that could affect the pathogenicity of the 2012 Mexican viruses, we analyzed the entire genomes of four isolates. Several non-synonymous substitutions as compared to the A/California/04/2009(H1N1) reference virus were detected in every segment (Table 2), with the exception of PA and M. Most of the substitutions had been previously reported in GenBank for a small number of samples from Asia, Oceania and the United States, during the 2010–2011 influenza season (Table 2). Pathogenesis-related mutations like E627K in PB2 [21], [22], D222G in HA [23], the PDZ ligand domain in NS [24] or N66S in PB1-F2 [25] were not detected in Mexican sequences. Only substitutions T20I in PB1 and S69T, K163R and N260D in HA were unique to the 2012 Mexican isolates. Nevertheless, their biological significance remains unknown. Additionally, all 2012 Mexican isolates had a histidine residue at position 274 of the NA gene (275 in N1 numbering), which is consistent with sensitivity to the neuraminidase inhibitor oseltamivir.

### Phylogenetic Analysis

**Figure 3:**
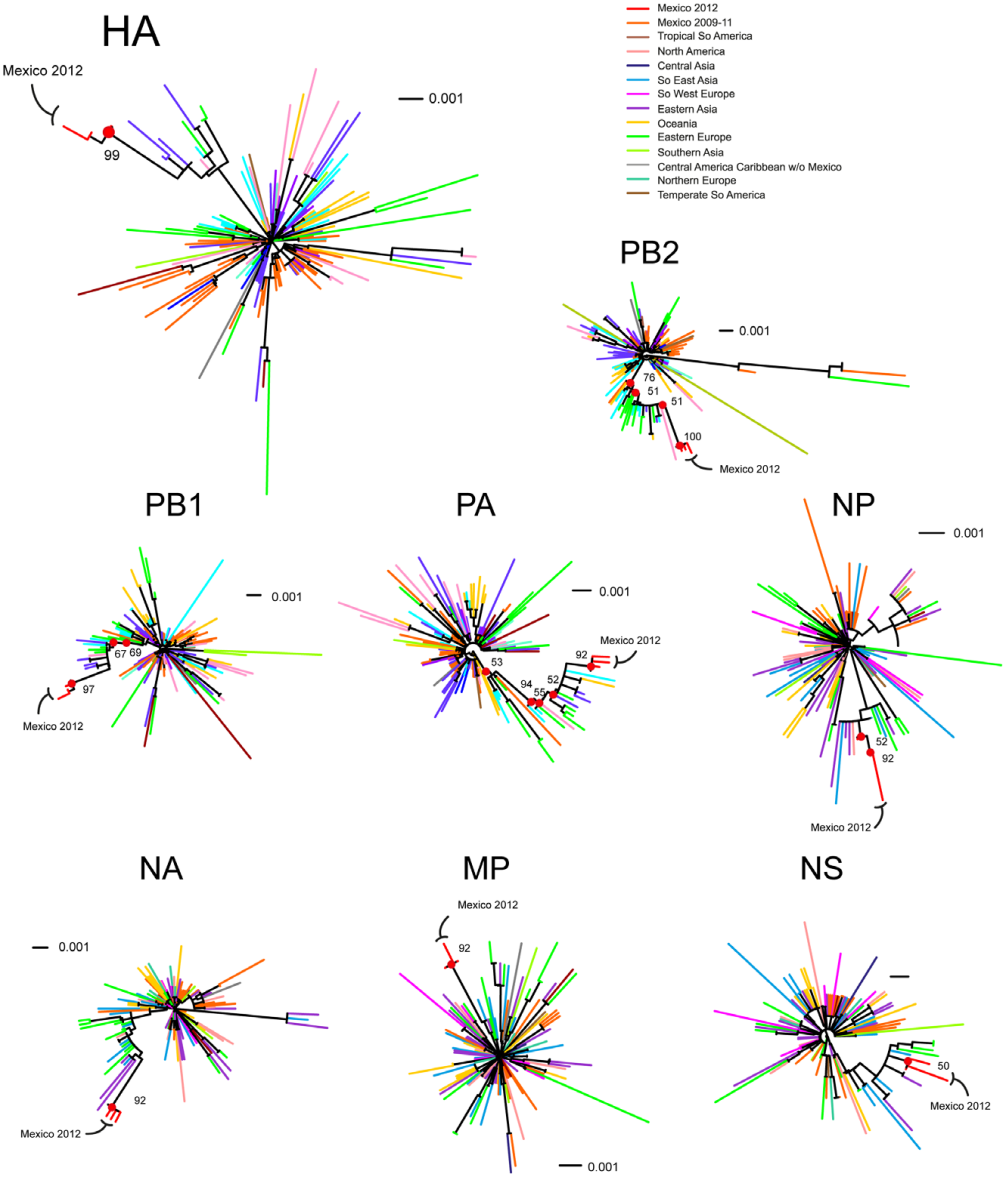
Maximum likelihood (ML) phylogenetic trees for the 8 influenza genetic segments. ML trees from 136 to 162 A(H1N1)pdm09 viruses registered in GenBank were produced with 1,000 bootstrap replicates, for the indicated genetic segments as explained in the Methods section. The four 2012 Mexican fully sequenced by next generation sequencing are included. Red dots at nodes show branches with >50% bootstrap support leading to the 2012 sequences described in this work. Branches are colored according to WHO influenza transmission zones [13].

Phylogenetic analysis based on the HA gene showed that Mexican influenza HA sequences collected in the 2011–2012 influenza season formed a robust distinct monophyletic group (bootstrap value of ≥99%, Figure 3). Mexican sequences clustered with HA sequences of strains isolated in East and South-East Asia (e.g., Thailand, Taiwan, and Ulaanbaatar) and sequences isolated in Eastern Europe (Russia), Australia (Sydney) and the USA (South Carolina) in 2010–2011 (Figure 3). Asian sequences within this cluster and 2012 Mexican sequences shared mutations S143G, S185T, E374K, S451N, and V520A not present in 2009 isolates. These mutations have been recently described as a genetic signature of viruses from the 2010–2011 influenza season in the United Kingdom [26], Hong Kong [27], Australia, Singapore and New Zealand [28]. Mutations A197T and S203T, present in 2012 Mexican isolates, had been previously reported in viruses from 2009 circulating in Japan [29], India [30], Russia [31] and the UK [32]. Remarkably, the substitutions S69T, K163R, and N260D were a unique signature to the 2012 Mexican sequence cluster. Sequences with the full set of mutations of these new variants have been reported exclusively in North America during 2012. Interestingly, 2012 Mexican HA sequences did not form clusters with 2009 Mexican HA sequences, suggesting different origins of the 2009 and the 2011–2012 outbreaks (Figure 3).

**Figure 4:**
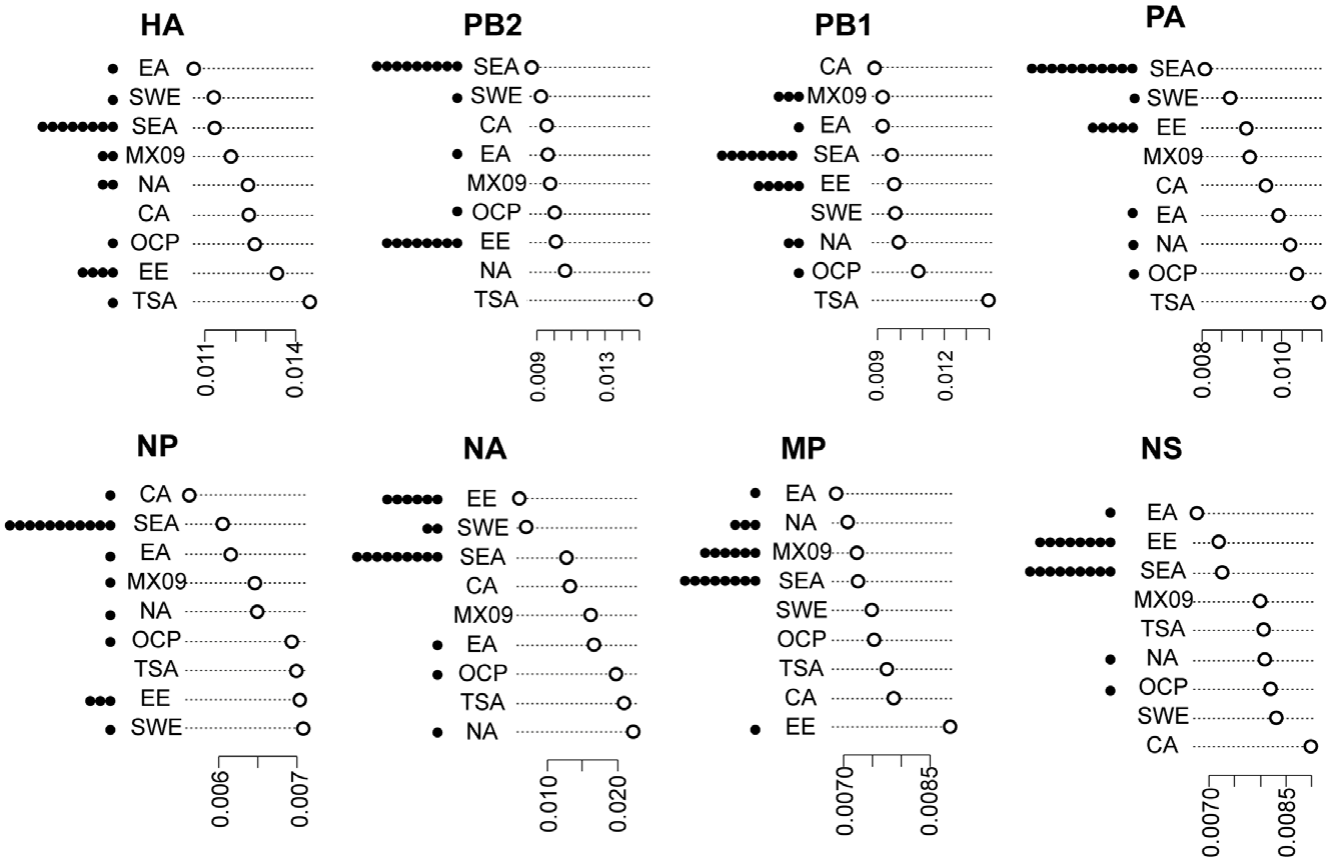
Genetic distances between Mexican 2012 isolates and viruses from all over the world. On the right side of the plot the average distance between the Mexican 2012 sequences and the sequences included for each geographical transmission zone [13] in Figure 3 is shown, for each influenza gene segment. On the left a histogram is shown with the distribution of the 20 closest sequences to the Mexican 2012 cluster for each viral segment. The four 2012 Mexican viruses sequenced by NGS are considered. A single virus from Central America and Caribbean and one from Central Asia were omitted from the distance graphs. SEA, South Eastern Asia; SWE, South Eastern Europe; EE, Eastern Europe; CA, Central Asia; OCP, Oceania, Micronesia and Polynesia; NA, North America; SA, Tempered South America; MX09, Mexican sequences from 2009 to 2011.

Phylogenetic analysis of each of the genetic segments showed similar clustering patterns. In all cases, 2012 Mexican isolates grouped together in highly significant clusters (Bootstrap value >90%, except for NP and NS, Figure 3). 2012 Mexican isolates were highly divergent compared to the rest of the isolates. For all segments, isolates mainly from East and South-East Asia (Ulaanbaatar, Taiwan, and Bangkok) and Eastern Europe (Russia) tended to group with 2012 Mexican Isolates (Figure 3), suggesting a common ancestor of the 2010–2011 Asian viruses and the 2011–2012 Mexican viruses. Evolutionary divergence analyses confirmed the lowest divergence between 2012 Mexican isolates and South-East Asian and East European sequences (Figure 4). As expected, 2012 Mexican sequences tended to cluster with more recent sequences, especially sequences from 2010–2011, than from 2009, and this was true for phylogenetic trees based on most of the genetic segments (Figure S2).

## Discussion

Following the post-pandemic period initiation in August 2010, predominance of the A(H1N1)pdm09 virus was observed for the first time in the 2011–2012 influenza season in Mexico and most countries of Central America. A previous European study found that during the reemergence of the A(H1N1)pdm09 virus there was an overrepresentation of severe cases within this group, with 20/34 (58.8%) of cases being severe, even though the total prevalence was less than 1% [5]. In the present study, among the positive samples, the prevalence of A(H1N1)pdm09 was 100%, which is similar to national data [3], [4] reporting a peak prevalence of A(H1N1)pdm09 of 94.76% during week 6, 2012. Of our confirmed cases, 82% required hospitalization, 21% required mechanical ventilation and 4% died, similar to what was found in Europe and at the national level in Mexico.

The 2011–2012 Mexican outbreak shared some clinical characteristics with the 2009 pandemic, including age of the patients and similar symptomatic presentation [33]. However, in the 2011–2012 outbreak, morbid obesity (identified as a risk factor for hospitalization in the 2009 pandemic [34]), and diabetes did not seem to be risk factors for severe presentation of the disease. The factors associated with the need of mechanical ventilation were delay in seeking medical attention, older age, and concomitant cardiovascular disease. During the study period, the percentage of nasopharyngeal swabs positive for influenza, all of them A(H1N1)pdm09, peaked at 80% and stabilized at 46%, possibly suggesting high levels of viral replication in the nasopharynx. The higher rate of detection of positive samples could be related to an earlier medical examination of patients and a better management of sampling and testing for the virus resulting in higher sensitivity. Indeed, nasal discharge/congestion was more prevalent in confirmed cases than in suspect cases (p = 0.04), which could indicate an earlier examination of patients with positive tests, coinciding with the onset of clinical symptoms.

Interestingly, approximately a fifth of all patients with ILI (18% of the confirmed cases), received previous influenza vaccination during the 2011–2012 influenza season, according to medical records. Moreover, the rates of viral isolation from vaccinated patients was higher than would be expected, according to previously reported vaccine effectiveness [17] and vaccination coverage in Mexico [18]. This observation argues against a full effectiveness of the vaccine in our setting and could be reflecting possible antigenic changes in the circulating viruses. Alternatively, this observation could also reflect vaccine-related issues such as lack of consistency in annual vaccination, or suboptimal responses to the vaccine. These observations have to be considered carefully, as an important proportion of the patients included in the present study were immunocompromised and/or presented with chronic lung disease (Table 1). Nevertheless, no difference was observed between the number of vaccinated immunocompromised and vaccinated immunocompetent confirmed cases (22.2% and 16.9% respectively, p = 0.60) arguing against a possible bias in vaccine effectiveness introduced by considering immunocompromised individuals.

In order to detect mutations in viral proteins that could influence antigenicity and viral pathogenicity, the entire genome of four viruses was analyzed by next generation sequencing. It is important to mention that the analyzed viruses were not cultured in order to avoid the generation of adaptive mutations *in vitro*. Remarkably, amino acid changes within or adjacent to the four HA antigenic sites in 2012 Mexican isolates were found (Figure 2). Three-dimensional modeling of the 2012 Mexican HA showed the accessibility of residues associated with antigenic sites and with changes with respect to the 2009 reference virus, suggesting a possible impact in the antigenicity of the protein. The presence of new mutations, together with other mutations previously observed in viruses of the 2010–2011 season, in or close to the HA antigenic sites warrants further antigenicity studies and is relevant for decision making on the H1 strain to be included in future vaccines [35].

As shown in Table 2, non-synonymous substitutions with respect to the prototype isolate H1N1 (A/California/04/2009) were detected in all viral segments, with the exception of PA. Indeed, phylogenetic analyses using the PA gene segment showed similar genetic divergence of the 2012 Mexican isolates and 2010–2011 Asian sequences, while other segments such as PB2, PB1, HA, and NA reflected a more unique divergence of the 2012 Mexican isolates. Most substitutions found in the 2012 Mexican isolates had been previously reported in GenBank, mainly during the 2010–2011 influenza season in Asia, Oceania and the USA, further linking 2012 Mexican isolates with Asian sequences of the 2010–2011 season. Importantly, the non-synonymous substitutions observed in the HA segment of the four viruses sequenced by NGS were also observed in the additional 8 viruses whose partial HA sequences were obtained by Sanger sequencing. Although the small number of samples analyzed in the present study is a limitation, finding the same novel mutations in all the viruses studied supports the argument that these mutations are a common feature of the Mexican 2011–2012 viruses.

Characteristic mutations of highly pathogenic influenza viruses, including E627K in PB2 of H5N1 and H7N7 viruses [21], [22], N66S in PB1-F2 of the 1918 H1N1 virus [25], D222G in HA of Pandemic A(H1N1)2009 [23], H274Y in NA of H5N1 viruses [36], or the PDZ ligand domain in the NS1 protein in the 1918 H1N1 and H5N1 viruses [24], were not present in 2012 Mexican isolates. Some unique substitutions to the 2012 Mexican viruses such as T20I in PB1 and S69T, K163R, N260D in HA were detected, confirming a unique genetic signature of this new viral cluster. However, the biological relevance of these mutations is still unknown.

Mexican 2011–2012 isolates formed a single genetic cluster characterized by novel substitutions as well as other substitutions already reported for the different viral proteins in isolates from the 2010–2011 influenza season. The 2012 Mexican isolates tended to group with Asian sequences in the phylogenetic analyses for most genetic segments, and no clustering with Mexican isolates from the 2009 pandemic was evident. Moreover, genetic distances were lowest between 2012 Mexican sequences and South-East Asian and East European sequences. These observations suggest that the reemerging influenza virus in Mexico might be derived from Asian viruses of the 2010–2011 season. Indeed the 2011–2012 and the 2009 Mexican viruses have a direct relation as the pandemic is believed to have originated with viruses that appeared in Mexico in 2009. However, our analyses suggest that the 2011–2012 Mexican viruses may derive from a reintroduction of the virus from Asian sources, rather than evolution of the 2009 viruses within Mexico. To the best of our knowledge, the new mutations described here have not been previously reported, suggesting that they originated in the viruses that gave rise to this specific outbreak.

Further studies are required to determine if the amino acid substitutions described here for the 2012 Mexican isolates may be related with the sudden predominance of H1N1 cases in Mexico, and if they represent an important antigenic drift which may enable some degree of viral immune evasion. A gradual drift in the A(H1N1)pdm09 virus was recently suggested by a study reporting an older age distribution of laboratory-confirmed A(H1N1)pdm09 influenza hospitalizations and deaths in Mexico during the 2011–2012 influenza season, relative to 2009 pandemic patterns [4], although our study did not confirm this. The impact of these changes on vaccine effectiveness in Mexican population has not been defined either. In February 2012 the World Health Organization recommended that the Northern Hemisphere's 2012–2013 seasonal influenza vaccine should include the A/California/7/2009 (H1N1)pdm09-like virus; the A/Victoria/361/2011 (H3N2)-like virus; and the B/Wisconsin/1/2010-like virus (from the B/Yamagata lineage of viruses) [35]. While the H1N1 virus is the same, the H3N2 and B vaccine viruses are different from those that were selected for the Northern Hemisphere for the 2011–2012 influenza vaccine. We suggest that the findings presented here should be considered in further tests for defining the H1N1 component of the seasonal influenza vaccine. Also, our findings contribute useful surveillance data on the evolution of the pandemic H1N1 viruses.

## Supporting Information

Figure S1 Three-dimensional modeling of HA from 2012 Mexican isolates. The three-dimensional structures of two HA proteins from 2012 Mexican isolates (HA2012a, and HA2012b, which harbors the K163R mutation) were predicted based on the crystal structure of a 2009 H1N1 influenza virus HA chain A [15] (PDB 3LZG), using the ESyPred3D web server [14]. The models are shown in three different orientations, one in each row of the figure. Antigenic sites are colored according to Figure 2: Sa – purple, Sb – yellow, Ca – green, Cb – blue. Changes in the 2012 Mexican isolates with respect to the 2009 reference virus are shown in red.
(TIF)

Figure S2 Comparison of phylogenetic trees built with the 8 influenza A genetic segments by date. Maximum likelihood genetic trees were produced for each influenza genetic segment with 1,000 bootstrap replicates as explained for Figure 4. A: PB2, B: PB1, C: PA, D: HA, E: NP, F: NA, G: MP, H: NS. Branches are colored according to date: yellow, 2011; green, 2010; blue, 2009. 2012 Mexican sequences are shown in red.
(TIFF)
