# Supplementary figures and images for: Molecular Characterization of the Predominant Influenza A(H1N1)pdm09 Virus in Mexico, December 2011–February 2012
Source: PLoS One. 2012 Nov 29;7(11):e50116. doi: 10.1371/journal.pone.0050116 (PMC3510220; doi:10.1371/journal.pone.0050116)

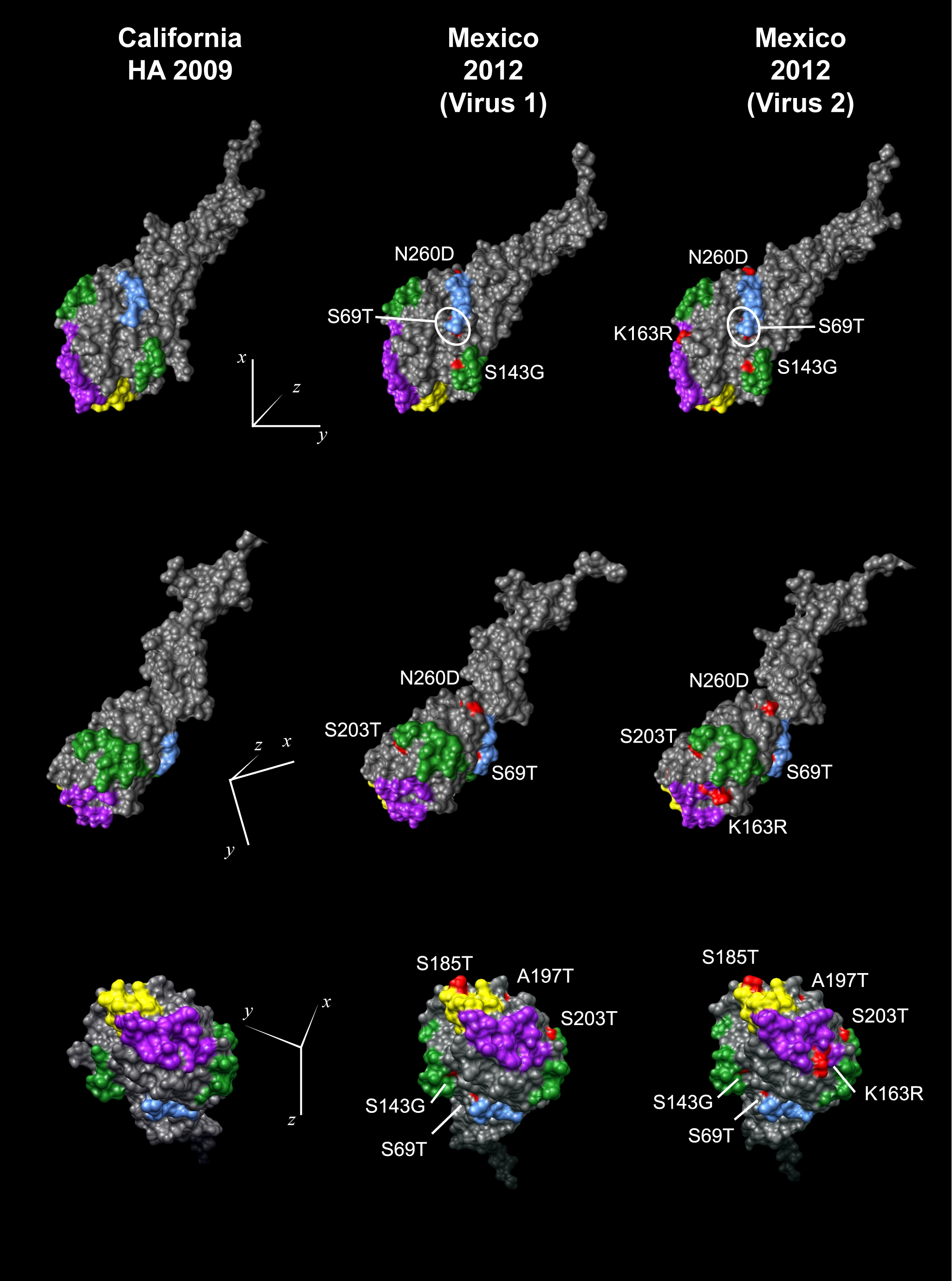

Supplement: Figure S1 — Three-dimensional modeling of HA from 2012 Mexican isolates. The three-dimensional structures of two HA proteins from 2012 Mexican isolates (HA2012a, and HA2012b, which harbors the K163R mutation) were predicted based on the crystal structure of a 2009 H1N1 influenza virus HA chain A [15] (PDB 3LZG), using the ESyPred3D web server [14]. The models are shown in three different orientations, one in each row of the figure. Antigenic sites are colored according to Figure 2: Sa – purple, Sb – yellow, Ca – green, Cb – blue. Changes in the 2012 Mexican isolates with respect to the 2009 reference virus are shown in red. (TIF) [file pone.0050116.s001.tif]

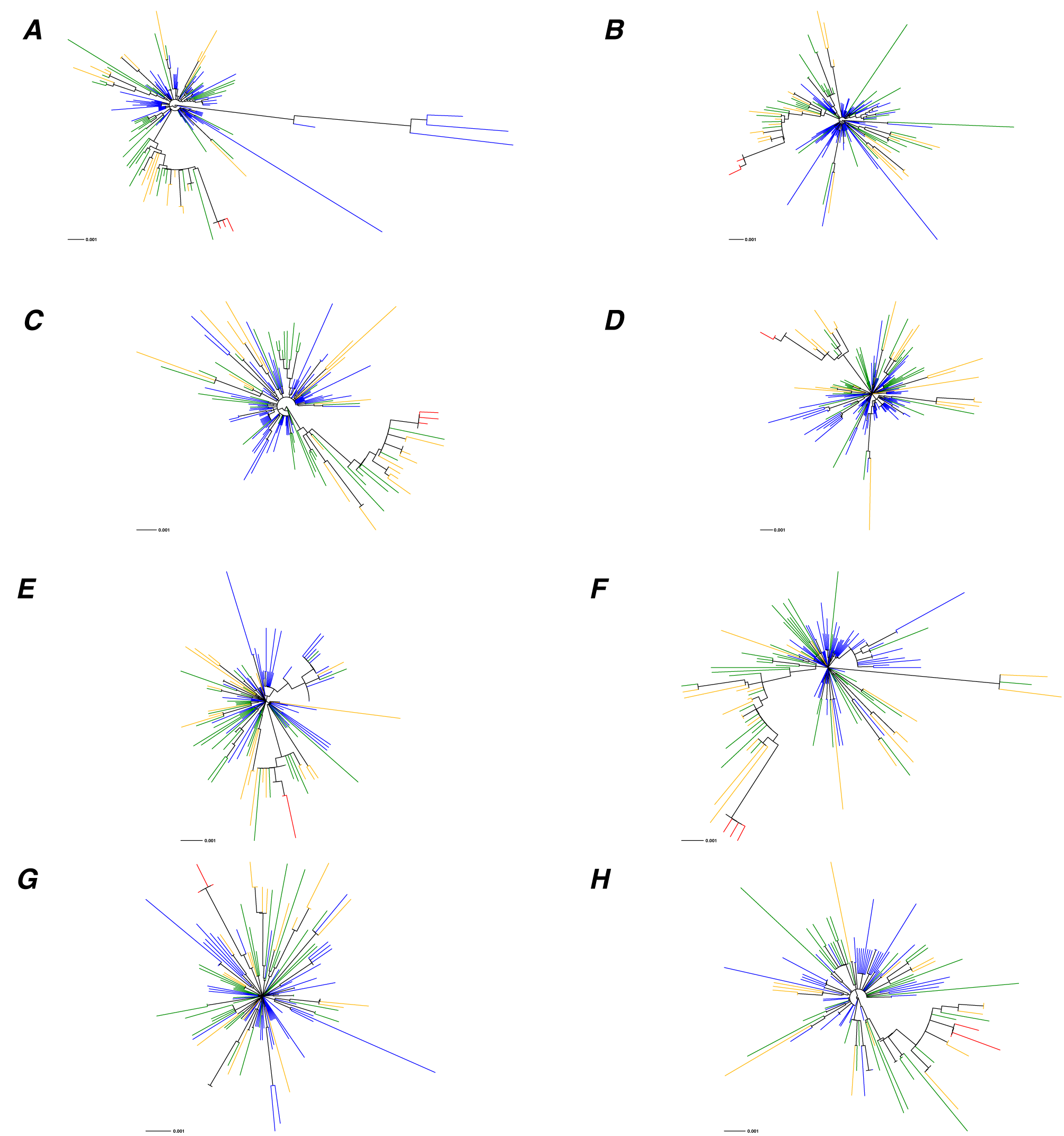

Supplement: Figure S2 — Comparison of phylogenetic trees built with the 8 influenza A genetic segments by date. Maximum likelihood genetic trees were produced for each influenza genetic segment with 1,000 bootstrap replicates as explained for Figure 4. A: PB2, B: PB1, C: PA, D: HA, E: NP, F: NA, G: MP, H: NS. Branches are colored according to date: yellow, 2011; green, 2010; blue, 2009. 2012 Mexican sequences are shown in red. (TIFF) [file pone.0050116.s002.tif]
